# Oxidative Stress in Diabetic Cardiomyopathy: Molecular Mechanisms and Emerging Therapeutic Targets

**DOI:** 10.3390/biom16030470

**Published:** 2026-03-20

**Authors:** Umberto Capece, Davide Nilo, Cassandra Morciano, Roberto Nilo, Serenella Spiezia, Marta Chiara Sircana, Vincenzo Russo, Marco Alfonso Perrone, Leonilde Bonfrate, Carlo Acierno, Ferdinando Carlo Sasso, Alfredo Caturano

**Affiliations:** 1 Centro Malattie Endocrine e Metaboliche, Dipartimento di Scienze Mediche e Chirurgiche, Fondazione Policlinico Universitario A. Gemelli IRCCS and Università Cattolica del Sacro Cuore, 00168 Roma, Italy; capeceumberto@gmail.com (U.C.); morcianocassandra@gmail.com (C.M.); 2 Department of Advanced Medical and Surgical Sciences, University of Campania “Luigi Vanvitelli”, 80138 Naples, Italy; nilodavide@gmail.com (D.N.); ferdinandocarlo.sasso@unicampania.it (F.C.S.); 3 Meditrial Europe, 00198 Roma, Italy; robertonilo98@gmail.com; 4 Diagnostic and Therapeutic Medicine Department, Fondazione Policlinico Universitario Campus Bio-Medico, Via Alvaro del Portillo, 200, 00128 Rome, Italy; serenella.spiezia@gmail.com; 5 Department of Medical, Surgical and Pharmacology, University of Sassari, 07100 Sassari, Italy; m.sircana4@phd.uniss.it; 6 Division of Cardiology, Department of Medical Translational Sciences, University of Campania “Luigi Vanvitelli”, 80138 Naples, Italy; v.p.russo@libero.it; 7 Department of Biology, College of Science and Technology, Sbarro Institute for Cancer Research and Molecular Medicine, Temple University, Philadelphia, PA 19122, USA; 8 Division of Cardiology and CardioLab, Department of Clinical Sciences and Translational Medicine, University of Rome Tor Vergata, 00133 Rome, Italy; marco.perrone@uniroma2.it; 9 Department of Human Sciences and Promotion of the Quality of Life, San Raffaele Roma University, 00166 Rome, Italy; leonilde.bonfrate@uniroma5.it; 10 Center of Nutrition for the Research and the Care of Obesity and Metabolic Diseases, National Institute of Gastroenterology IRCCS “Saverio de Bellis”, 70013 Castellana Grotte, Italy; 11 Azienda Ospedaliera Regionale San Carlo, 85100 Potenza, Italy; carlo894@gmail.com

**Keywords:** diabetic cardiomyopathy, oxidative stress, mitochondrial dysfunction, reactive oxygen species, epicardial adipose tissue, myocardial fibrosis, redox signaling, antioxidant defense, heart failure, diabetes mellitus

## Abstract

Diabetic cardiomyopathy (DCM) is a distinct myocardial disorder that develops independently of coronary artery disease and hypertension and represents a major contributor to heart failure in patients with diabetes. Beyond hemodynamic alterations, DCM is driven by complex molecular mechanisms involving metabolic dysregulation, mitochondrial dysfunction, inflammation, and fibrotic remodeling. Increasing evidence identifies oxidative stress as a central integrative process linking these pathogenic pathways in the diabetic heart. Chronic hyperglycemia, insulin resistance, and altered substrate utilization promote excessive generation of reactive oxygen species, overwhelming endogenous antioxidant defenses and disrupting myocardial redox homeostasis. Oxidative stress induces direct damage to lipids, proteins, and DNA while simultaneously activating redox-sensitive signaling pathways that amplify inflammation, endothelial dysfunction, cardiomyocyte apoptosis, and fibrosis. In addition, epicardial and visceral adipose tissue have emerged as active contributors to myocardial oxidative stress through paracrine and systemic mechanisms, reinforcing inflammatory and fibrotic crosstalk. This review provides a comprehensive overview of the molecular sources and targets of oxidative damage in DCM, examines the impairment of antioxidant defense systems, and discusses emerging therapeutic strategies aimed at restoring redox balance.

## 1. Introduction

Diabetes mellitus represents a major global health burden and is one of the leading causes of cardiovascular morbidity and mortality worldwide [1]. In addition to accelerated atherosclerosis and ischemic heart disease, diabetes is associated with a distinct myocardial disorder known as diabetic cardiomyopathy (DCM), characterized by structural and functional alterations of the myocardium that occur independently of coronary artery disease, hypertension, or valvular abnormalities [2,3]. Clinically, DCM manifests as progressive left ventricular dysfunction, initially dominated by diastolic impairment with preserved ejection fraction and, in advanced stages, by systolic dysfunction and overt heart failure. Despite its clinical relevance and growing prevalence, DCM remains underdiagnosed and lacks disease-specific therapeutic strategies [3].

The pathogenesis of diabetic cardiomyopathy is complex and multifactorial, reflecting the chronic metabolic stress imposed by hyperglycemia, insulin resistance, and altered substrate utilization. These disturbances lead to profound changes in myocardial energy metabolism, lipid handling, and cellular homeostasis [4]. Rather than acting as independent mechanisms, these processes converge on oxidative stress, which has emerged as a central and unifying driver of myocardial injury in the diabetic heart [5]. Persistent metabolic overload promotes excessive generation of reactive oxygen species (ROS), overwhelming endogenous antioxidant defenses and disrupting redox balance across multiple cardiac cell types [6]. Oxidative stress plays a dual pathogenic role in DCM. On the one hand, excess ROS directly damage lipids, proteins, and nucleic acids, impairing cellular integrity and contractile function. On the other hand, oxidative stress acts as a key signaling mediator that amplifies maladaptive pathways involved in inflammation, fibrosis, and cell death [7]. Through activation of redox-sensitive kinases and transcription factors, oxidative stress contributes to endothelial dysfunction, microvascular impairment, cardiomyocyte apoptosis, and fibroblast activation, thereby promoting adverse myocardial remodeling and progressive ventricular dysfunction [7,8,9,10].

Beyond cardiomyocytes and endothelial cells, increasing evidence highlights the contribution of extracardiac tissues to myocardial oxidative stress in diabetes. In particular, epicardial adipose tissue (EAT) and other visceral fat depots have emerged as active pathogenic players through paracrine and systemic mechanisms [11,12]. In the diabetic milieu, adipose tissue dysfunction, macrophage infiltration, and altered adipokine secretion further intensify local inflammation and oxidative burden within the cardiac microenvironment [6]. These observations support the concept that DCM is not a purely cardiomyocyte-centric disease but rather the result of a complex multicellular and inter-organ crosstalk driven by metabolic and redox imbalance. Despite substantial progress in delineating the molecular mechanisms underlying DCM, current clinical management largely relies on glycemic control and conventional heart failure therapies, which do not specifically target the underlying myocardial pathology [4,13]. This limitation has fueled growing interest in oxidative stress and redox-regulated pathways as potential disease-modifying targets, with the aim of interrupting the progression of myocardial damage at earlier stages.

The aim of this review is to provide a comprehensive overview of the role of oxidative stress in the development and progression of diabetic cardiomyopathy. Specifically, we (i) summarize the key pathophysiological features of DCM, (ii) discuss the major cellular sources and molecular targets of oxidative damage in the diabetic heart, (iii) explore the interplay between oxidative stress, inflammation, and fibrotic remodeling, and (iv) highlight emerging therapeutic strategies and future perspectives aimed at restoring redox homeostasis and improving cardiac outcomes in patients with diabetes. Unlike reviews that focus on individual molecular pathways or isolated antioxidant mechanisms, this article provides an integrated synthesis that positions oxidative stress as a central organizing process of this condition. In addition, we will focus on recent evidence particularly regarding adipose tissue, which makes an update of this nosological entity necessary. Furthermore, most studies in the current literature focus on the effects of GLP-1 receptor agonists (GLP-1RA) [14] and SGLT2 inhibitors (SGLT2-I) [15], which act on diabetic cardiomyopathy through multiple mechanisms that are still under investigation and mainly independent of oxidative stress. Our objective, instead, is to focus specifically on this pathogenetic mechanism. This review emphasizes the emerging shift from non-specific antioxidant supplementation toward precision, mechanism-based redox therapies that target specific sources, intracellular compartments, and disease stages. This integrated framework aims to support a more mechanistic and translational understanding of redox-targeted interventions in diabetic cardiomyopathy.

## 2. Pathophysiology of Diabetic Cardiomyopathy

DCM represents a distinct pathological entity that differs from other forms of heart failure by its unique metabolic, structural, and molecular features. Unlike ischemic or hypertensive cardiomyopathy, DCM develops in the absence of overt coronary artery disease or pressure overload and is primarily driven by chronic metabolic stress associated with diabetes, which converges on sustained oxidative stress as a central mediator of myocardial injury [4,16]. The hallmark of DCM is a progressive impairment of myocardial structure and function, initially characterized by diastolic dysfunction with preserved ejection fraction and subsequently evolving toward systolic dysfunction and overt heart failure [17].

At the structural level, diabetic hearts exhibit a constellation of histopathological changes, including cardiomyocyte hypertrophy, interstitial and perivascular fibrosis, microvascular rarefaction, and increased cardiomyocyte apoptosis. These alterations lead to increased myocardial stiffness, impaired relaxation, and reduced ventricular compliance, which are key determinants of diastolic dysfunction [16]. Fibrotic remodeling, in particular, reflects excessive deposition of extracellular matrix components, predominantly collagen types I and III, driven by persistent activation of cardiac fibroblasts and profibrotic signaling pathways [18]. Over time, this maladaptive remodeling compromises not only mechanical efficiency but also electrical stability of the myocardium [19,20].

From a metabolic standpoint, the diabetic heart undergoes a profound shift in substrate utilization. Insulin resistance and reduced glucose uptake force cardiomyocytes to rely predominantly on fatty acid oxidation for ATP generation [21]. While fatty acids constitute an efficient energy source under physiological conditions, their chronic overutilization in diabetes results in mitochondrial overload, incomplete β-oxidation, and accumulation of toxic lipid intermediates such as ceramides and diacylglycerols [22,23]. This metabolic inflexibility not only reduces cardiac energetic efficiency but also promotes lipotoxicity, mitochondrial dysfunction, and excessive generation of ROS, thereby directly linking altered substrate metabolism to myocardial redox imbalance [24,25].

Mitochondrial abnormalities are a central feature of DCM pathophysiology and represent a critical source of oxidative stress. Diabetic cardiomyocytes display altered mitochondrial morphology, impaired oxidative phosphorylation, reduced ATP production, and enhanced electron leak from the respiratory chain [26]. These defects amplify ROS generation and initiate a cascade of downstream events, including mitochondrial DNA damage, opening of the mitochondrial permeability transition pore, activation of apoptotic pathways, and disruption of calcium handling [27,28]. Impaired calcium homeostasis further contributes to contractile dysfunction and increases arrhythmogenic susceptibility, reinforcing the progression toward heart failure [29]. In parallel, diabetes induces profound alterations in myocardial signaling networks. Chronic hyperglycemia, insulin resistance, and oxidative stress activate redox-sensitive pathways such as protein kinase C (PKC), mitogen-activated protein kinases (MAPKs), nuclear factor-κB (NF-κB), and transforming growth factor-β (TGF-β) [30]. These pathways orchestrate inflammatory responses, promote fibroblast activation, and sustain maladaptive myocardial remodeling. Importantly, oxidative stress functions as a key amplifier of these signaling cascades, integrating metabolic derangements with inflammation, fibrosis, and cardiomyocyte loss [31,32].

Collectively, these structural, metabolic, and molecular alterations define the complex pathophysiological landscape of diabetic cardiomyopathy. Rather than resulting from a single pathogenic insult, DCM emerges from the convergence of metabolic inflexibility, mitochondrial dysfunction, oxidative stress, inflammation, and fibrotic remodeling. Within this framework, oxidative stress should be viewed not merely as a downstream consequence of diabetes but as a central integrative mechanism linking these processes. This concept provides the rationale for a focused analysis of the cellular and molecular sources of ROS in diabetic cardiomyopathy, which are discussed in the following section.

## 3. Sources of Oxidative Stress in Diabetic Cardiomyopathy

### 3.1. Cellular Types Involved

The development of oxidative stress in DCM involves multiple cardiac and extracardiac cell types, including cardiomyocytes, endothelial cells of the coronary microvasculature, cardiac fibroblasts, and adipocytes from epicardial and other visceral fat depots [6,12,16]. Cardiomyocytes are directly exposed to hyperglycemia, insulin resistance, and altered substrate metabolism, leading to mitochondrial dysfunction, impaired calcium handling, and disruption of intracellular redox balance [32]. Endothelial cells respond to the diabetic milieu with increased ROS production, reduced nitric oxide (NO) bioavailability, and endothelial dysfunction, contributing to microvascular impairment [33]. In parallel, EAT has emerged as an active contributor to myocardial oxidative stress. Epicardial adipocytes can release free fatty acids, pro-inflammatory cytokines, and adipokines that act locally through paracrine signaling or systemically via the circulation, thereby promoting oxidative stress, inflammation, and fibrotic remodeling within the myocardium [12,34,35]. Adipose depots located outside the heart may further amplify this process by supplying circulating pro-oxidant and pro-inflammatory mediators [36]. The coordinated dysfunction of these cellular compartments results in a sustained oxidative burden that drives myocardial injury and functional deterioration in DCM.

### 3.2. Mitochondrial Dysfunction and ROS Overproduction

Mitochondrial dysfunction represents a primary source of oxidative stress in DCM. In diabetic cardiomyocytes, mitochondria exhibit marked morphological and functional abnormalities, including fragmentation, altered fission–fusion dynamics that may involve MFN1/2, OPA1, and DRP1, impaired electron transport chain efficiency, and increased electron leakage [37,38,39]. All these processes ultimately lead to excessive superoxide production [39,40]. Hyperglycemia and fatty-acid overload increase the supply of reducing equivalents (NADH and FADH_2_) to the respiratory chain, further enhancing mitochondrial ROS generation and overwhelming intramitochondrial antioxidant systems such as manganese superoxide dismutase and mitochondrial catalase [40]. This redox imbalance induces mitochondrial DNA (mtDNA) damage, opening of the mitochondrial permeability transition pore, cytochrome c release, and activation of apoptotic pathways, thereby contributing to cardiomyocyte loss, contractile dysfunction, and fibrotic remodeling [37,41]. Importantly, mitochondrial ROS generation establishes a self-perpetuating cycle, whereby oxidative damage further impairs mitochondrial function and amplifies ROS production [38,39]. In addition to electron leak from respiratory chain complexes, cytochrome c has been identified as a context-dependent contributor to ROS generation. Under physiological conditions, cytochrome c functions as a mobile electron carrier between complexes III and IV [42]. However, in the presence of elevated NADH and hydrogen peroxide concentrations, conditions frequently observed in the diabetic myocardium, cytochrome c may participate in redox cycling reactions capable of generating superoxide radicals [43,44]. Moreover, partial release of cytochrome c from mitochondria during early mitochondrial membrane destabilization can further disrupt electron transport chain efficiency, amplifying ROS production [45]. This dual role of cytochrome c, both as an essential component of oxidative phosphorylation and as a potential amplifier of oxidative stress under pathological conditions, underscores the complexity of mitochondrial redox regulation in diabetic cardiomyopathy.

### 3.3. NADPH Oxidases (NOX Enzymes)

In addition to mitochondria, NADPH oxidases (NOX) constitute a major enzymatic source of ROS in DCM. NOX isoforms (particularly NOX2, NOX4, and NOX5) are expressed in cardiomyocytes, endothelial cells, and cardiac fibroblasts. In the diabetic setting, NOX activity is stimulated by hyperglycemia, activation of diacylglycerol-protein kinase C pathways, angiotensin II signaling, and inflammatory mediators [46,47]. NOX-derived ROS promote superoxide formation, reduce NO bioavailability, and activate downstream inflammatory cascades, including NF-κB signaling, thereby amplifying oxidative injury and myocardial inflammation [25,48]. Experimental studies suggest that selective inhibition of NOX enzymes may attenuate oxidative stress and pathological remodeling in DCM [49].

### 3.4. Xanthine Oxidoreductase and Aldehyde Oxidase

Xanthine oxidoreductase (XOR) represents an additional enzymatic source of reactive oxygen species in the diabetic heart. XOR exists in two interconvertible forms, xanthine dehydrogenase and xanthine oxidase, the latter preferentially generating superoxide anion and hydrogen peroxide during purine metabolism [50]. Under physiological conditions, XOR activity is tightly regulated; however, in pathological states such as diabetes mellitus, ischemia–reperfusion injury, and chronic inflammation, increased expression and post-translational conversion to the oxidase form enhance myocardial ROS production [7,10,51]. In patients with type 2 diabetes, elevated plasma xanthine oxidoreductase activity has been significantly associated with insulin resistance, adverse metabolic parameters, and vascular complications, supporting the concept that XOR-derived oxidative stress may contribute to endothelial dysfunction and cardiovascular risk in the diabetic milieu [50,52].

Aldehyde oxidase (AO), a molybdenum-containing enzyme structurally related to XOR, has also emerged as a potential contributor to cardiac oxidative stress [53]. Although primarily studied in hepatic metabolism, AO is expressed in cardiovascular tissues and is capable of generating superoxide and hydrogen peroxide during oxidation of endogenous aldehydes and xenobiotics [54,55]. In diabetic conditions, increased availability of reactive aldehydes and enhanced inflammatory signaling [56] may favor greater AO-dependent redox activity, potentially contributing to additional intracellular ROS generation. Although direct evidence in diabetic cardiomyopathy remains limited, the biochemical properties of aldehyde oxidase support a plausible contributory role under conditions of metabolic and inflammatory stress, thus representing a still insufficiently characterized source of ROS in this context.

### 3.5. Uncoupled Nitric Oxide Synthase (NOS)

Endothelial nitric oxide synthase (eNOS) normally produces NO, exerting vasodilatory and cardioprotective effects. However, under conditions of oxidative stress, hyperglycemia, and deficiency of essential cofactors such as tetrahydrobiopterin (BH_4_), eNOS may become uncoupled, shifting from NO production to superoxide generation. This process further reduces NO bioavailability and exacerbates oxidative damage within both the endothelium and myocardium [10,41]. Experimental studies in diabetic models have demonstrated eNOS uncoupling in vascular tissues and endothelial progenitor cells, characterized by increased superoxide production and impaired endothelial function [57,58]. These alterations contribute to endothelial dysfunction and impaired NO signaling, mechanisms widely recognized as key contributors to diabetic cardiovascular complications, including diabetic cardiomyopathy [10,41]. Although eNOS uncoupling is well documented in vascular and endothelial contexts, its direct contribution to myocardial oxidative injury in DCM remains incompletely defined and should be interpreted as an extrapolated mechanism.

### 3.6. Advanced Glycation End-Products (AGEs) and RAGE Signaling

Chronic hyperglycemia promotes the formation and accumulation of advanced glycation end-products (AGEs), which interact with their receptor RAGE to activate pro-oxidant and pro-inflammatory signaling pathways. AGE–RAGE engagement enhances ROS generation through stimulation of NADPH oxidases, activates NF-κB signaling, and induces expression of inflammatory and fibrotic genes [59,60]. In the diabetic myocardium, this axis significantly contributes to oxidative stress accumulation, myocardial fibrosis, and ventricular dysfunction [61].

### 3.7. Endoplasmic Reticulum Stress and the Unfolded Protein Response (UPR)

Endoplasmic reticulum (ER) stress represents an additional contributor to oxidative stress in DCM [37]. Hyperglycemia, accumulation of misfolded or glycated proteins, and increased metabolic demand activate the unfolded protein response (UPR), mediated by PERK/eIF2α, IRE1α, and CHOP signaling pathways. Persistent UPR activation promotes ROS generation and exacerbates intracellular redox imbalance [37,62]. In diabetic cardiomyocytes, ER stress contributes to apoptosis, secondary mitochondrial dysfunction, and interstitial fibrosis, further reinforcing the oxidative and structural remodeling characteristic of DCM [63].

## 4. Molecular Targets of Oxidative Damage in the Heart

### 4.1. Lipids: Lipid Peroxidation and Membrane Damage

Excessive ROS production drives lipid peroxidation of membrane lipids, generating reactive aldehydes such as 4-hydroxy-2-nonenal (4-HNE) and malondialdehyde (MDA), which impair membrane fluidity, receptor function, ion-channel behavior and ion-pump activity [64,65]. In DCM, elevated MDA and 4-HNE levels have been correlated with the severity of myocardial dysfunction [66]. Damage to mitochondrial or plasma membranes of cardiomyocytes directly undermines cell integrity, compromises signal transduction and activates apoptotic pathways [32].

### 4.2. Proteins: Carbonylation and Enzymatic Inactivation

ROS can oxidize amino-acid residues (e.g., lysine, arginine, proline) leading to formation of carbonyl groups that alter protein tertiary structure, enzyme activity and protein stability [67]. The marker 3-nitrotyrosine (3-NT) reflects protein nitration by peroxynitrite and is elevated in cardiomyopathies [68]. Within DCM, protein carbonylation contributes to dysfunction of contractile proteins, ion-pump proteins and mitochondrial proteins, thereby disturbing energetic metabolism and increasing susceptibility to apoptosis [66].

### 4.3. DNA: Mitochondrial and Nuclear Damage

ROS can damage mitochondrial DNA (mtDNA) and nuclear DNA, causing single-strand or double-strand breaks, base oxidation (e.g., 8-oxo-2′-deoxyguanosine, 8-OH-dG) and activation of DNA-repair pathways [69,70,71]. In diabetic hearts, increased mtDNA damage has been documented and correlates with mitochondrial dysfunction and cardiac fibrosis [38,72]. DNA damage promotes p53 activation, cardiomyocyte senescence and apoptosis, thus contributing to DCM progression toward heart failure [73].

### 4.4. Redox-Sensitive Signaling Pathways

Oxidative stress activates several redox-sensitive signaling cascades that play a central role in translating molecular damage into maladaptive cellular responses. Among these, MAPKs, including ERK1/2, p38, and JNK, are critically involved in the regulation of cardiomyocyte hypertrophy, apoptosis, and fibrotic responses. Sustained activation of these pathways in the diabetic myocardium promotes adverse structural remodeling and functional impairment [74,75].

The transcription NF-κB represents a key downstream effector of oxidative stress. ROS-mediated NF-κB activation induces the expression of pro-inflammatory cytokines, adhesion molecules, and extracellular matrix-related genes, thereby establishing a molecular link between oxidative injury and inflammatory priming of the myocardium [76]. Importantly, NF-κB signaling acts as an upstream trigger that initiates inflammatory and fibrotic programs, which are further amplified through cellular crosstalk and tissue-level responses [77] discussed in subsequent sections. In contrast to these pro-oxidant pathways, nuclear factor erythroid 2-related factor 2 (Nrf2) serves as the master regulator of the endogenous antioxidant response. Upon activation, Nrf2 translocates to the nucleus and induces the transcription of antioxidant and cytoprotective genes involved in ROS detoxification and redox homeostasis. However, in diabetic conditions, Nrf2 signaling is often impaired or insufficiently activated, resulting in reduced antioxidant capacity and increased vulnerability of cardiomyocytes to oxidative damage [78,79]. The imbalance between persistent activation of pro-oxidant signaling pathways (MAPKs and NF-κB) and inadequate activation of compensatory antioxidant mechanisms mediated by Nrf2 facilitates the progression from oxidative stress to sustained myocardial injury. This molecular dysregulation primes inflammatory and fibrotic responses, thereby contributing to myocardial remodeling and functional decline in diabetic cardiomyopathy [80].

**Figure 1:**
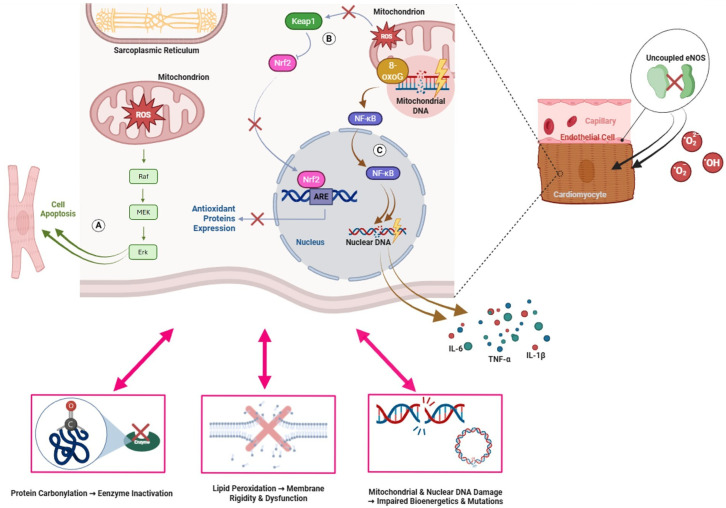
Molecular targets and intracellular consequences of oxidative damage in diabetic cardiomyopathy. Excessive mitochondrial production of reactive oxygen species (ROS) in cardiomyocytes induces oxidative damage to multiple intracellular targets. ROS activate pro-apoptotic signaling pathways, including the Raf–MEK–ERK cascade (**A**), and promote oxidative lesions in mitochondrial and nuclear DNA, such as 8-oxo-7,8-dihydroguanine (8-oxoG), leading to impaired bioenergetics and genomic instability. Oxidative stress disrupts the Keap1–Nrf2 axis, reducing antioxidant response element (ARE)-dependent transcription of antioxidant enzymes and weakening endogenous defense mechanisms (**B**). In parallel, ROS activate NF-κB signaling and induce transcriptional programs that exacerbate inflammatory responses and nuclear DNA damage (**C**). Uncoupling of endothelial nitric oxide synthase (eNOS) further contributes to ROS accumulation and endothelial dysfunction, amplifying oxidative injury in adjacent cardiomyocytes. At the molecular level, oxidative stress results in protein carbonylation with enzyme inactivation, lipid peroxidation leading to membrane rigidity and dysfunction, and cumulative mitochondrial and nuclear DNA damage that ultimately compromise cardiomyocyte survival and function [32,38,64,65,66,67,68,69,70,71,72,73,74,75,76,77,78,79,80].

Collectively, oxidative damage to lipids, proteins, and nucleic acids, together with the dysregulation of redox-sensitive signaling pathways, translates molecular redox imbalance into maladaptive cellular responses in the diabetic myocardium. These intracellular mechanisms are tightly interconnected and converge to impair cardiomyocyte survival, contractile function, and stress resilience. A schematic overview of the principal molecular targets and intracellular consequences of oxidative stress in diabetic cardiomyopathy is provided in Figure 1.

## 5. Inflammatory and Fibrotic Crosstalk

The pathophysiology of diabetic cardiomyopathy involves, beyond the myocardium itself, a complex interaction with other organs and tissues through the release of hormones, cytokines, and circulating metabolic mediators. In recent years, increasing attention has been directed toward the role of EAT as a key contributor to myocardial inflammation and oxidative stress. EAT is anatomically contiguous with the myocardium and is distributed along the atrioventricular and interventricular grooves surrounding the coronary arteries (peri-coronary EAT) and overlying the myocardial surface (myocardial EAT) [81,82]. Due to its anatomical position, EAT shares the same microcirculation as the myocardium, in the absence of interposed muscle fascia or connective tissue layers [83]. Consequently, alterations in EAT physiology can directly influence myocardial structure and function.

Under physiological conditions, EAT exerts several cardioprotective effects. It acts as a local energy reservoir, supplying free fatty acids to the myocardium, and secretes adipokines with anti-inflammatory and anti-atherogenic properties [84]. Moreover, EAT exhibits features of both white and brown adipose tissue, indicating a metabolically active phenotype [85]. However, under pathological conditions such as obesity and diabetes mellitus, EAT undergoes marked structural and functional remodeling [86]. EAT thickness increases and a phenotypic shift toward a pro-inflammatory and pro-fibrotic state occurs, characterized by enhanced secretion of cytokines such as IL-6, TNF-α, and MCP-1, together with reduced release of protective mediators including adiponectin [87,88,89]. Importantly, this transition reflects not merely an expansion of adipose mass but a profound metabolic and structural reprogramming of EAT [90]. Mitochondrial dysfunction in EAT enhances local oxidative stress and promotes the accumulation of lipotoxic intermediates, which may influence the adjacent myocardium through paracrine mechanisms facilitated by the absence of a fascial barrier and the shared microcirculation between these tissues, although direct evidence in DCM remains limited [81,90]. These paracrine signals may promote oxidative stress, endothelial activation, and recruitment of pro-inflammatory macrophages within the myocardial and perivascular compartments [91]. The resulting inflammatory microenvironment induces cardiomyocyte stress and interstitial remodeling, ultimately favoring myocardial fibrosis [92]. Persistent cytokine exposure and altered lipid handling from dysfunctional EAT further promote lipotoxicity and oxidative stress, contributing to increased myocardial stiffness, diastolic dysfunction, and heart failure with preserved ejection fraction [93]. Owing to its direct contact with atrial and ventricular myocardium, EAT dysfunction may also alter local electrophysiological properties, creating a substrate for atrial and ventricular arrhythmias and increasing the incidence of atrial fibrillation [94,95,96].

The mechanisms underlying the development of a dysfunctional, pro-inflammatory EAT phenotype are not fully elucidated. Under physiological conditions, EAT exhibits beige-like features and retains a certain thermogenic potential. However, in the diabetic milieu, EAT may undergo a phenotypic shift toward a white adipocyte-dominant profile (“EAT whitening”), characterized by reduced thermogenic capacity, mitochondrial dysfunction, and impaired oxidative efficiency [85,94]. Specifically, this process consists of the transformation of mitochondria-rich, multilocular brown adipocytes into large, unilocular white adipocytes [97]. During sustained caloric excess, whitened adipocytes undergo to increased triglyceride storage [98] and progressive hypertrophy until a critical size threshold is reached [99], beyond which cellular stress and adipocyte death occur [100]. This critical size is lower in visceral fat depots, such as EAT, compared with subcutaneous adipose tissue [101]. Adipocyte death promotes macrophage accumulation and the formation of crown-like structures, further amplifying local inflammation [102]. Moreover, macrophage density is generally higher in visceral adipose tissue, reinforcing its pro-inflammatory profile [103]. Collectively, chronic low-grade inflammation, macrophage polarization, and lipotoxic infiltration contribute to fibrotic remodeling, microvascular impairment, and electrical instability, thereby linking EAT dysfunction to adverse myocardial structural and functional changes in diabetic cardiomyopathy, although direct mechanistic evidence remains limited and this association is largely supported by indirect and translational data. These processes are highly interconnected and likely overlap in both diabetes and obesity [104]. Supporting this concept, EAT from patients with type 2 diabetes exhibits distinct transcriptional profiles characterized by increased expression of inflammatory genes and cytokines, reinforcing the view of EAT as an active pathogenic mediator rather than a passive fat depot [105].

Pharmacological interventions such as GLP-1 receptor agonists and SGLT2 inhibitors have demonstrated promising effects in reducing EAT thickness and improving cardiovascular health. These effects are likely mediated by indirect mechanisms related to weight loss and systemic metabolic improvement for SGLT2 inhibitors, and by both direct and indirect actions for GLP-1 receptor agonists, given the expression of GLP-1 receptors on EAT and their ability to modulate adipose tissue metabolism [106,107,108]. The cardioprotective properties of these agents are well recognized, and attenuation of EAT-derived oxidative stress may contribute to their beneficial cardiovascular effects [109].

Beyond EAT, other visceral adipose depots, including perirenal, supraclavicular, and paravertebral brown adipose tissue, may influence myocardial structure and function through analogous mechanisms involving adipose whitening, macrophage infiltration, and cytokine release [110,111]. These depots have been linked to cardiovascular health, suggesting that inflammatory and fibrotic crosstalk in diabetic cardiomyopathy likely involves multiple visceral fat compartments, among which EAT exerts the most pronounced effects due to its anatomical proximity to the heart [112]. Through direct paracrine signaling [113] and systemic dissemination of pro-inflammatory mediators from distant fat depots [114], adipose tissue dysfunction generates a “distal inflammatory storm” that reinforces myocardial oxidative stress. This systemic-local axis establishes a self-perpetuating cycle of oxidative injury, accelerating the transition from metabolic compensation to overt heart failure [115].

Within this oxidative and inflammatory milieu, a central role is played by the NLRP3 inflammasome, which functions as a redox-sensitive sensor in cardiomyocytes, fibroblasts, and endothelial cells [116]. Chronic hyperglycemia-induced metabolic stress promotes NLRP3 overactivation [117], leading to caspase-1 activation, pyroptosis, and excessive release of IL-1β and IL-18 [118,119]. These cytokines propagate inflammation and stimulate fibroblast activation and collagen deposition, thereby linking inflammatory signaling to fibrotic remodeling of the diabetic heart [120]. IL-1β also interacts with transforming growth factor-β (TGF-β) pathways, promoting extracellular matrix synthesis and altering fibroblast contractile activity [121]. TGF-β signaling mobilizes Smad2 and Smad3 transcription factors, promoting the expression of fibrosis-related genes and contributing to extracellular matrix accumulation [122]. Reactive oxygen species further amplify this process by enhancing TGF-β activation [123]. In parallel, activation of the NLRP3 inflammasome contributes to endothelial dysfunction, as IL-1β promotes endothelial-to-mesenchymal transition and loss of barrier integrity, thereby exacerbating inflammation and fibrotic remodeling [124]. These mechanisms position NLRP3 as a critical molecular link between metabolic stress, inflammation, and fibrosis in the diabetic heart. Moreover, sustained ROS generation driven by mitochondrial dysfunction and NADPH oxidases activate TGF-β signaling, enhance matrix-remodeling enzyme expression, and promote fibroblast proliferation and differentiation [123,125]. ROS further exacerbate AGE formation and RAGE activation, contributing to extracellular matrix accumulation [126]. Counter-regulatory mechanisms such as sirtuin-1 activation may attenuate oxidative stress, improve mitochondrial function, and promote adipose tissue browning, representing potential modulators of these pathogenic processes [123,127,128,129,130].

**Figure 2:**
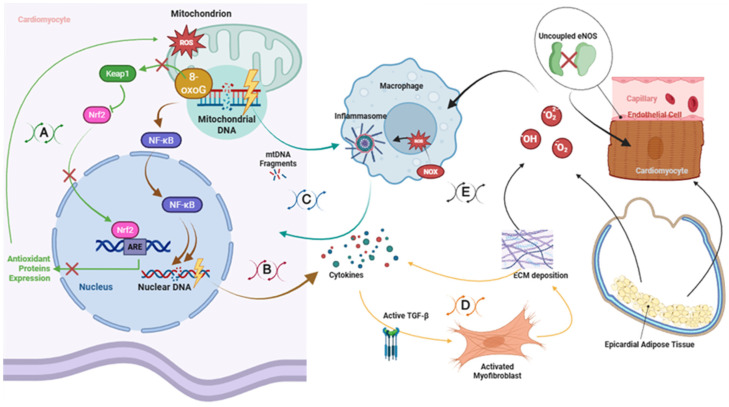
Vicious cycles linking oxidative stress, inflammation, and fibrosis in diabetic cardiomyopathy. (**A**) Oxidative DNA damage–antioxidant imbalance loop. Mitochondrial reactive oxygen species (ROS) induce accumulation of 8-oxo-7,8-dihydroguanine (8-oxoG) in mitochondrial and nuclear DNA, impairing mitochondrial function and further increasing ROS production. Excess ROS also disrupts the Keap1–Nrf2 axis, reducing antioxidant response element (ARE)-dependent expression of antioxidant enzymes (e.g., SOD, GPx, catalase, HO-1), thereby reinforcing oxidative stress. (**B**) NF-κB–cytokine–DNA damage loop. ROS and metabolic stress activate NF-κB signaling, leading to increased expression of pro-inflammatory cytokines such as IL-6 and TNF-α. Cytokine signaling enhances nuclear oxidative damage and further NF-κB activation, establishing a self-amplifying inflammatory circuit. (**C**) mtDNA–NLRP3 inflammasome loop. Oxidatively damaged mitochondrial DNA (mtDNA) is released as damage-associated molecular patterns (DAMPs), activating NADPH oxidase-derived ROS production and the NLRP3–caspase-1 inflammasome in macrophages. This promotes IL-1β and IL-18 secretion, which feeds back to cardiomyocytes, exacerbating mitochondrial dysfunction and ROS generation. (**D**) Cytokine–TGF-β–fibrosis loop. IL-1β, IL-18, TNF-α, and ROS activate latent transforming growth factor-β (TGF-β), driving fibroblast-to-myofibroblast differentiation and excessive extracellular matrix (ECM) deposition. Progressive fibrotic remodeling increases mechanical stress and local inflammation, sustaining cytokine release and tissue stiffening. (**E**) Integrated extrinsic drivers. Uncoupled endothelial nitric oxide synthase (eNOS), epicardial adipose tissue-derived cytokines and free fatty acids, macrophage NADPH oxidase activity, and paracrine ROS converge on cardiomyocytes and stromal cells, amplifying oxidative stress, inflammatory signaling, and fibrotic progression [81,82,83,84,85,86,87,88,89,90,91,92,93,94,95,96,97,98,99,100,101,102,103,104,105,106,107,108,109,110,111,112,113,114,115,116,117,118,119,120,121,122,123,124,125,126,127,128,129,130].

Overall, inflammatory and fibrotic crosstalk in diabetic cardiomyopathy arises from the integration of metabolic stress, oxidative imbalance, adipose tissue dysfunction, and redox-sensitive inflammatory signaling (Figure 2). These interconnected mechanisms contribute to myocardial remodeling, microvascular dysfunction, and progressive ventricular impairment, highlighting oxidative stress as a central driver linking metabolic disease to cardiac structural and functional deterioration.

## 6. Antioxidant Defense Mechanisms and Their Impairment

In physiological conditions, antioxidant defenses comprise a tightly regulated network of enzymatic and non-enzymatic systems that neutralize ROS and preserve cellular redox homeostasis. The main enzymatic antioxidants include superoxide dismutase (SOD), glutathione peroxidase (GPx), and catalase. SOD catalyzes the conversion of superoxide into hydrogen peroxide, which is subsequently detoxified by catalase and GPx, thereby preventing propagation of harmful oxidative cascades. In diabetes, persistent hyperglycemia and mitochondrial overproduction of ROS chronically activate pro-oxidant pathways and progressively impair the activity of these enzymes. As a result, SOD, catalase, and GPx often become insufficient or dysfunctional in diabetic tissues, contributing to sustained oxidative stress [131,132,133].

This enzymatic deficit is not merely a consequence of increased antioxidant consumption but reflects a deeper disruption of the cellular transcriptional antioxidant network governed by specific proteins, particularly Nrf2 and Nrf1 [134]. Nrf2 is the master regulator of the antioxidant response element pathway. Upon activation by oxidative stress, Nrf2 dissociates from Keap1, translocates to the nucleus, and induces the expression of multiple antioxidant and cytoprotective genes involved in ROS scavenging and redox balance [135]. In diabetes, impaired Nrf2 signaling is associated with reduced induction of these defense genes and increased susceptibility of cardiomyocytes and vascular cells to ROS-mediated injury [136].

Although less extensively studied, Nrf1 also plays a relevant role in maintaining redox homeostasis by regulating subsets of antioxidant genes under basal conditions [137]. Experimental evidence suggests that Nrf1 and Nrf2 exert cooperative and inter-regulatory functions in fine-tuning the expression of phase II detoxifying enzymes and ROS-scavenging systems. For instance, Nrf2 activation can promote Nrf1 transcription, while loss of either factor alters antioxidant gene expression and exacerbates oxidative stress. In addition, Nrf1 influences proteostasis and mitochondrial function, which are critical for cellular resilience against oxidative insults [138,139].

Beyond enzymatic systems, several non-enzymatic antioxidants contribute to myocardial redox control. Reduced glutathione (GSH) and coenzyme Q10 (CoQ10) directly scavenge free radicals and support enzymatic antioxidant recycling [140]. In diabetes, intracellular GSH pools are frequently depleted due to increased oxidative consumption and impaired synthesis or regeneration, thereby sustaining oxidative stress and inflammation. CoQ10, a lipophilic antioxidant and key component of the mitochondrial electron transport chain, is similarly affected; reduced tissue or circulating levels and impaired redox cycling have been reported in individuals with diabetes. Randomized trials and meta-analyses suggest that CoQ10 supplementation may improve selected metabolic and oxidative markers, although heterogeneity among studies and limited bioavailability constrain definitive conclusions [141,142].

Additional modulators of antioxidant defense further link metabolic status to redox homeostasis. Irisin, a myokine highly expressed in cardiomyocytes, enhances SIRT1 expression and has been shown to restore reduced GSH levels typically observed in diabetic cardiomyopathy [143]. High-density lipoproteins (HDLs) also exert anti-inflammatory and anti-fibrotic effects in the myocardium [144]: apolipoprotein A-1 inhibits the production of IL-1β and TNF-α [145] while promoting expression of the anti-inflammatory cytokine IL-10 [146]. Moreover, HDL downregulates angiotensin II type 1 receptor expression, leading to reduced NAD(P)H oxidase activity and lower ROS generation [147]. However, hyperglycemia-induced lipid peroxidation promotes the formation of oxidized and dysfunctional HDL, thereby limiting these protective effects [148]. Finally, cellular metabolic state can influence antioxidant capacity. Moderate elevations in circulating D-β-hydroxybutyrate have been shown to enhance resistance to oxidative stress by restoring thioredoxin and GPx4 expression [149]. These observations suggest that metabolic substrate shifts, such as partial reliance on ketone bodies, may help reinforce antioxidant defenses and mitigate oxidative injury in diabetic cardiomyopathy.

## 7. Emerging Therapeutic Strategies

Growing evidence indicates that the limited clinical success of conventional antioxidant therapies in diabetic cardiomyopathy does not undermine the pathogenic role of oxidative stress per se but rather reflects the inadequacy of non-specific reactive oxygen species scavenging strategies. Oxidative stress in the diabetic heart is not merely the result of excessive ROS accumulation but arises from a complex dysregulation of spatially and temporally controlled redox signaling pathways that are essential for cellular adaptation and survival. Accordingly, contemporary therapeutic approaches have shifted from indiscriminate antioxidant supplementation toward mechanism-based strategies aimed at selectively modulating redox homeostasis at its primary sources and signaling nodes. These emerging interventions target mitochondrial ROS production, NADPH oxidase activity, redox-sensitive transcriptional programs, and organelle-specific oxidative signaling, with the goal of restoring physiological redox balance while preserving adaptive redox signaling [17,19]. Within this evolving framework, redox-targeted therapies are increasingly viewed as precision interventions capable of modifying disease progression rather than simply neutralizing oxidative by-products.

### 7.1. Mitochondria- and Enzyme-Targeted Redox Modulation

Excessive production of ROS, particularly from mitochondria and NOX, represents a major contributor to oxidative injury in the diabetic heart [49,150]. Persistent oxidative stress promotes lipid, protein, and DNA damage, mitochondrial dysfunction, inflammation, cardiomyocyte apoptosis, and myocardial fibrosis, ultimately leading to adverse ventricular remodeling and functional impairment [6,49,151]. Importantly, the limited or inconsistent efficacy observed with classical antioxidant supplementation in clinical studies, including vitamins C and E, has highlighted fundamental limitations of non-targeted ROS scavenging strategies [151,152]. These approaches may fail to reach critical intracellular compartments, interfere with physiological redox signaling, or be administered at stages when irreversible myocardial damage has already occurred. Consequently, the lack of clinical benefit should not be interpreted as evidence against the pathogenic role of oxidative stress in diabetic cardiomyopathy but rather as an indication that oxidative stress must be targeted with greater mechanistic precision [153,154,155,156]. This realization has driven the development of contemporary redox-based therapies aimed at selectively modulating ROS production and redox-sensitive pathways at their primary cellular sources. Pharmacological antioxidants have therefore been investigated as potential strategies to counteract this redox imbalance. In contrast to conventional antioxidants that primarily neutralize ROS downstream of their formation, mitochondria-targeted redox therapies are designed to act directly at the primary intracellular source of pathological ROS generation [157]. By selectively accumulating within mitochondria, agents such as MitoQ, MitoTEMPO, and SS-31 (elamipretide) directly modulate mitochondrial redox balance, preserve electron transport chain function, and interrupt self-amplifying cycles of oxidative damage at their origin [158,159]. MitoQ, a coenzyme Q analog conjugated to a lipophilic cation, has shown efficacy in preclinical models of diabetes; thus, further studies are needed to confirm these findings in functional models of the heart [158,159]. Similarly, MitoTEMPO has been demonstrated to selectively scavenge mitochondrial superoxide, improving endothelial function and attenuating cardiomyocyte apoptosis and hypertrophy in experimental models of both type 1 and type 2 diabetes [160]. Tempol, a membrane-permeable superoxide dismutase mimetic, has been shown to reduce superoxide accumulation, preserve nitric oxide bioavailability, and improve myocardial redox balance, resulting in cardioprotective effects in diabetic cardiovascular dysfunction [161]. In addition, apocynin, a NOX inhibitor, has been extensively studied in diabetic animal models, where it reduced cardiac hypertrophy and fibrosis, improved mitochondrial function, restored antioxidant enzyme activity, and attenuated adverse ventricular remodeling by limiting NOX-derived ROS production [162,163,164].

Despite encouraging preclinical data, translation of pharmacological antioxidants into clinical practice remains limited. Variability in bioavailability, lack of tissue specificity, and the complexity of redox signaling may account for the modest or inconsistent benefits observed in clinical studies, underscoring the need for more targeted and mechanism-based approaches.

### 7.2. Natural Compounds and Nutraceuticals

Natural compounds and nutraceuticals have attracted interest not merely for their intrinsic antioxidant properties but primarily for their ability to modulate redox-sensitive signaling pathways involved in mitochondrial function, inflammation, and metabolic regulation [165,166]. Polyphenolic compounds such as resveratrol and quercetin enhance mitochondrial biogenesis through activation of SIRT1 and AMP-activated protein kinase (AMPK), reduce oxidative damage, and improve calcium handling in cardiomyocytes [130,167]. Curcumin, derived from *Curcuma longa*, exerts cardioprotective effects by inhibiting NF-κB signaling, reducing advanced glycation end-product formation, and attenuating myocardial fibrosis [168]. Alpha-lipoic acid (ALA) functions as a redox modulator capable of regenerating endogenous antioxidants such as glutathione and vitamin C, while also improving endothelial function and insulin sensitivity [169]. Although these compounds demonstrate promising biological effects, clinical evidence remains heterogeneous. Differences in formulation, dosage, bioavailability, and study design limit definitive conclusions, highlighting the need for standardized clinical trials specifically addressing diabetic cardiomyopathy.

### 7.3. Looking to the Future: Gene Therapy and Redox Enzyme Modulation

Targeting redox-sensitive transcription factors and enzymatic systems involved in ROS regulation represents a promising strategy to correct the underlying oxidative imbalance in DCM. These approaches aim to restore redox homeostasis and re-establish adaptive cellular responses disrupted in the diabetic heart [154,170]. Modulation of Nrf2, NOX enzymes, and SIRT1 should therefore be viewed in future as an approach to re-establish physiological redox homeostasis, rather than as a form of exogenous antioxidant supplementation [8,47,135]. Activation of Nrf2 induces expression of antioxidant enzymes such as heme oxygenase-1 and glutathione peroxidase, conferring protection against oxidative myocardial injury [171]. Conversely, inhibition of NOX isoforms, particularly NOX2 and NOX4, has been shown to prevent cardiomyocyte apoptosis and pathological remodeling [172]. We have already discussed the effects of sirtuin-1 (SIRT1), a NAD^+^-dependent deacetylase, on GSH levels. The modulation of SIRT1 has also shown to attenuate oxidative stress and exert protective effects in the diabetic heart [173]. Gene-based strategies aimed at enhancing these protective pathways may offer sustained correction of redox imbalance, although safety and long-term effects remain important challenges.

### 7.4. Lifestyle Interventions

Lifestyle interventions remain a cornerstone in the management of diabetes and exert significant effects on myocardial redox homeostasis. Caloric restriction reduces systemic inflammation, lowers circulating Fetuin-A levels, and downregulates Toll-like receptor signaling, thereby improving cardiac metabolism and contractility [174]. Regular physical activity enhances myocardial antioxidant enzyme activity, improves calcium handling, and increases mitochondrial efficiency [175]. In addition, strict glycemic control attenuates ROS generation and limits advanced glycation end-product accumulation, slowing the progression of myocardial fibrosis [176]. These interventions provide systemic benefits that synergize with pharmacological therapies.

### 7.5. Advanced Therapeutic Approaches

Recent advances in nanotechnology and peptide-based therapies have opened up new avenues for targeted redox modulation. Nanocarriers enable precise delivery of antioxidants, peptides, or microRNAs to specific tissues or subcellular compartments, overcoming limitations of conventional antioxidants such as poor bioavailability and off-target effects [177,178,179]. Various platforms, including liposomes, dendrimers, biodegradable polymers, and bioinspired calcium phosphate nanoparticles, have been explored for cardiac applications [180]. Mitochondria-targeted peptides such as SS-31 (elamipretide) bind cardiolipin within the inner mitochondrial membrane, stabilizing the electron transport chain, reducing ROS production, and improving ATP synthesis. In experimental models of diabetic cardiomyopathy, SS-31 has been shown to attenuate fibrosis, reduce cardiac remodeling, and improve ventricular function [179]. The combination of organellar targeting with multifunctional nanocarriers may further enhance therapeutic efficacy while minimizing systemic toxicity, representing a promising direction for future precision medicine approaches [158,179,181]. Although these advanced strategies remain largely preclinical, they offer compelling prospects for selectively targeting the molecular drivers of oxidative stress in diabetic cardiomyopathy.

Collectively, these emerging strategies reflect a conceptual shift from non-specific antioxidant supplementation toward precision redox medicine in diabetic cardiomyopathy. Increasing evidence indicates that therapeutic efficacy depends on targeting the appropriate redox source, intracellular compartment, and disease stage, rather than indiscriminate ROS neutralization [8,154]. Moreover, interindividual heterogeneity in metabolic status, disease duration, and myocardial phenotype suggests that redox-targeted interventions may require patient stratification and biomarker-guided timing to achieve meaningful clinical benefit [17,150,182]. This evolving framework underscores the need to integrate mechanistic specificity, temporal intervention, and patient phenotyping when translating redox-based therapies into clinical practice. The main redox-targeted therapeutic strategies, their molecular targets, and the current level of experimental and clinical evidence are summarized in Table 1.

## 8. Challenges and Future Directions

Despite significant advances in elucidating the molecular mechanisms underlying DCM, several critical challenges continue to limit the effective translation of experimental findings into clinical practice. One of the major obstacles lies in the substantial gap between preclinical models and human disease. Most mechanistic insights into oxidative stress-driven myocardial injury derive from animal models or in vitro systems that only partially recapitulate the metabolic complexity, disease duration, and comorbidity burden observed in patients with diabetes [183,184]. As a result, therapeutic strategies, particularly those targeting redox imbalance, that demonstrate robust efficacy in experimental settings often fail to show consistent clinical benefits.

Another important challenge is the marked heterogeneity of DCM. In fact, DCM encompasses a broad spectrum of phenotypes, ranging from early diastolic dysfunction with preserved ejection fraction to advanced systolic failure, influenced by diabetes duration, glycemic control, obesity, sex, genetic background, and concomitant cardiovascular risk factors [16,185]. This heterogeneity suggests that a “one-size-fits-all” approach to redox-targeted therapies is unlikely to be effective. Future strategies should therefore move toward personalized and phenotype-driven interventions, potentially guided by metabolic, inflammatory, and oxidative stress profiles.

The identification and validation of reliable biomarkers of oxidative stress represent an additional unmet need. Currently available circulating markers often lack specificity for myocardial redox imbalance and are influenced by systemic inflammation and comorbid conditions [17]. Integration of redox-related biomarkers with advanced imaging modalities, such as cardiac magnetic resonance and positron emission tomography, may improve early detection of subclinical myocardial involvement and enable monitoring of disease progression and therapeutic response.

Emerging omics-based approaches, including transcriptomics, proteomics, metabolomics, and epigenomics, offer promising tools to unravel the complex molecular networks linking metabolism, oxidative stress, inflammation, and fibrosis in DCM and potentially drive precision medicine treatments [186,187]. However, their clinical utility will depend on integration with redox-specific biomarkers and functional cardiac endpoints, rather than on isolated molecular signatures. Systems biology and machine-learning-based analyses may help identify key regulatory nodes and stratify patients according to dominant pathogenic mechanisms, thereby refining therapeutic targeting [188]. Finally, future research should prioritize well-designed clinical trials specifically addressing diabetic cardiomyopathy, rather than extrapolating data from broader heart failure populations. Such trials should incorporate mechanistic endpoints related to oxidative stress, biomarker-driven patient stratification, and long-term follow-up to determine whether redox-targeted interventions can meaningfully alter the natural history of DCM.

## 9. Conclusions

DCM is a complex myocardial disorder that develops independently of coronary artery disease and hypertension and represents a major contributor to heart failure in patients with diabetes. Accumulating evidence supports oxidative stress as a central integrative mechanism linking metabolic dysregulation, mitochondrial dysfunction, inflammation, and fibrotic remodeling in the diabetic heart.

Multiple cellular sources of reactive oxygen species, including dysfunctional mitochondria, NADPH oxidases, uncoupled nitric oxide synthase, advanced glycation end-product signaling, and endoplasmic reticulum stress, converge to disrupt myocardial redox homeostasis. This imbalance promotes oxidative damage to lipids, proteins, and DNA, activates maladaptive signaling pathways, and drives progressive structural and functional deterioration of the myocardium. In parallel, epicardial and visceral adipose tissue have emerged as active contributors to myocardial oxidative stress through paracrine and systemic mechanisms, reinforcing inflammatory and fibrotic crosstalk. Although effective disease-specific therapies for diabetic cardiomyopathy are still lacking, targeting oxidative stress and redox-regulated pathways represents a promising strategy for disease modification. Advances in pharmacological antioxidants, nutraceuticals, gene-based approaches, lifestyle interventions, and targeted delivery systems provide new opportunities to restore redox balance and preserve myocardial function. However, successful clinical translation will require improved biomarkers, refined patient stratification, and well-designed clinical trials to determine whether redox-targeted interventions can meaningfully alter the course of diabetic cardiomyopathy.

## Figures and Tables

**Table 1:** Redox-targeted therapeutic strategies in diabetic cardiomyopathy: molecular targets and level of evidence.

Therapeutic Strategy	Representative Agents/Approaches	Primary Molecular Targets	Mechanism of Redox Modulation	Evidence	Refs.
Mitochondria-targeted redox therapies	MitoQ, MitoTEMPO, SS-31 (elamipretide), Tempol	Mitochondrial electron transport chain, cardiolipin	Direct modulation of mitochondrial ROS production or catalytic detoxification of superoxide and related radicals; preservation of electron transport chain function and interruption of self-amplifying oxidative damage cycles	Preclinical (robust); early clinical (SS-31)	[159,160,161,162,179]
NOX inhibition	Apocynin, NOX2/NOX4 inhibitors	NADPH oxidase isoforms	Source-specific reduction in ROS generation and attenuation of redox-driven inflammatory and fibrotic signaling	Preclinical	[162,163,164,165,172]
Redox-sensitive transcriptional reprogramming	Nrf2 activation strategies	Keap1–Nrf2 axis	Restoration of endogenous antioxidant and cytoprotective gene expression; re-establishment of redox homeostasis	Preclinical	[8,171]
Metabolic–redox regulators	SIRT1 activation (e.g., resveratrol), AMPK modulation	SIRT1, AMPK pathways	Recalibration of redox-sensitive metabolic signaling, mitochondrial biogenesis, and stress resistance	Preclinical; limited clinical	[130,167,173]
Nutraceutical redox modulators	Resveratrol, curcumin, α-lipoic acid	NF-κB, SIRT1, AMPK, endogenous antioxidant systems	Modulation of redox-sensitive inflammatory and metabolic pathways rather than direct ROS scavenging	Preclinical; heterogeneous clinical	[130,167,168,169]
Advanced delivery systems	Nanocarriers, mitochondria-targeted peptides	Subcellular redox compartments	Improved tissue and organelle specificity of redox-targeted interventions	Preclinical	[177,178,179,180,181]

Abbreviations: reactive oxygen species (ROS), mitochondrial electron transport chain (ETC), NADPH oxidase (NOX), nuclear factor erythroid 2-related factor 2 (Nrf2), sirtuin-1 (SIRT1), AMP-activated protein kinase (AMPK), nuclear factor-κB (NF-κB), mitochondria-targeted ubiquinone (MitoQ), mitochondria-targeted superoxide dismutase mimetic (MitoTEMPO), mitochondria-targeted peptide SS-31 (elamipretide).

## Data Availability

No dataset was generated for the publication of this article.

## Abbreviations

The following abbreviations are used in this manuscript:

AGEs	Advanced glycation end-products
ALA	Alpha-lipoic acid
AMPK	AMP-activated protein kinase
ARE	Antioxidant response element
ATP	Adenosine triphosphate
BH_4_	Tetrahydrobiopterin
CoQ10	Coenzyme Q10
DCM	Diabetic cardiomyopathy
DRP1	Dynamin-related protein 1
EAT	Epicardial adipose tissue
eNOS	Endothelial nitric oxide synthase
ER	Endoplasmic reticulum
ERK	Extracellular signal-regulated kinase
FADH_2_	Flavin adenine dinucleotide (reduced form)
GPx	Glutathione peroxidase
GSH	Reduced glutathione
HDL	High-density lipoprotein
HFpEF	Heart failure with preserved ejection fraction
IL	Interleukin
IRE1α	Inositol-requiring enzyme 1 alpha
JNK	c-Jun N-terminal kinase
Keap1	Kelch-like ECH-associated protein 1
MAPKs	Mitogen-activated protein kinases
MDA	Malondialdehyde
MFN	Mitofusin
mtDNA	Mitochondrial DNA
NADH	Nicotinamide adenine dinucleotide (reduced form)
NADPH	Nicotinamide adenine dinucleotide phosphate
NF-κB	Nuclear factor kappa-light-chain-enhancer of activated B cells
NO	Nitric oxide
NOX	NADPH oxidase
Nrf1	Nuclear factor erythroid 2-related factor 1
Nrf2	Nuclear factor erythroid 2-related factor 2
OPA1	Optic atrophy protein 1
PERK	Protein kinase RNA-like endoplasmic reticulum kinase
PKC	Protein kinase C
RAGE	Receptor for advanced glycation end-products
ROS	Reactive oxygen species
SIRT1	Sirtuin-1
SOD	Superoxide dismutase
TGF-β	Transforming growth factor beta
TNF-α	Tumor necrosis factor alpha
UPR	Unfolded protein response
4-HNE	4-hydroxy-2-nonenal
8-OH-dG	8-hydroxy-2′-deoxyguanosine
